# Serum-based measurements of stromal activation through ADAM12 associate with poor prognosis in colorectal cancer

**DOI:** 10.1186/s12885-022-09436-0

**Published:** 2022-04-12

**Authors:** Sanne ten Hoorn, Cynthia Waasdorp, Martijn G. H. van Oijen, Helene Damhofer, Anne Trinh, Lan Zhao, Lisanne J. H. Smits, Sanne Bootsma, Gabi W. van Pelt, Wilma E. Mesker, Linda Mol, Kaitlyn K. H. Goey, Miriam Koopman, Jan Paul Medema, Jurriaan B. Tuynman, Inti Zlobec, Cornelis J. A. Punt, Louis Vermeulen, Maarten F. Bijlsma

**Affiliations:** 1 Amsterdam UMC location University of Amsterdam, Center for Experimental and Molecular Medicine, Laboratory for Experimental Oncology and Radiobiology, Cancer Center Amsterdam, Imaging and Biomarkers, Meibergdreef 9, Amsterdam, the Netherlands; 2 Cancer Center Amsterdam, Imaging and Biomarkers, Amsterdam, the Netherlands; 3 Oncode Institute, Amsterdam, The Netherlands; 4 Amsterdam UMC location University of Amsterdam, Department of Medical Oncology, Cancer Center Amsterdam, Meibergdreef 9, Amsterdam, The Netherlands; 5 Cell Biology Program, Memorial Sloan Kettering Cancer Center, New York, USA; 6 Department of Medical Oncology, Dana-Farber Cancer Institute, Boston, USA; 7 Department of Electronic Engineering, City University of Hong Kong, Kowloon, Hong Kong; 8 Amsterdam UMC location Vrije Universiteit Amsterdam, Department of Surgery, Cancer Center Amsterdam, Boelelaan 1117, Amsterdam, the Netherlands; 9 Department of Surgery, Leiden University Medical Center, Leiden, The Netherlands; 10 Department of Data Management, Netherlands Comprehensive Cancer Center (IKNL), Nijmegen, The Netherlands; 11 Department of Medical Oncology, University Medical Center Utrecht, Utrecht University, Utrecht, The Netherlands; 12 Institute of Pathology, University of Bern, Bern, Switzerland; 13 Department of Epidemiology, Julius Center for Health Sciences and Primary Care, University Medical Center, Utrecht University, Utrecht, The Netherlands

**Keywords:** Colorectal cancer, Prognostic marker, ADAM12, Stroma

## Abstract

**Background:**

Recently it has been recognized that stromal markers could be used as a clinically relevant biomarker for therapy response and prognosis. Here, we report on a serum marker for stromal activation, A Disintegrin and Metalloprotease 12 (ADAM12) in colorectal cancer (CRC).

**Methods:**

Using gene expression databases we investigated *ADAM12* expression in CRC and delineated the source of ADAM12 expression. The clinical value of ADAM12 was retrospectively assessed in the CAIRO2 trial in metastatic CRC with 235 patients (31% of total cohort), and an independent rectal cancer cohort (*n* = 20).

**Results:**

ADAM12 is expressed by activated CRC associated fibroblasts. In the CAIRO2 trial cohort, ADAM12 serum levels were prognostic (ADAM12 low versus ADAM12 high; median OS 25.3 vs. 17.1 months, HR 1.48 [95% CI 1.11–1.96], *P* = 0.007). The prognostic potential was specifically high for metastatic rectal cancer (HR 1.78 [95% CI 1.06–3.00], *P* = 0.030) and mesenchymal subtype tumors (HR 2.12 [95% CI 1.25–3.60], *P* = 0.004). ADAM12 also showed potential for predicting recurrence in an exploratory analysis of non-metastatic rectal cancers.

**Conclusions:**

Here we describe a non-invasive marker for activated stroma in CRC which associates with poor outcome, especially for primary cancers located in the rectum.

**Supplementary Information:**

The online version contains supplementary material available at 10.1186/s12885-022-09436-0.

## Background

Colorectal cancer (CRC) is currently the second leading cause of cancer-related deaths worldwide [1]. Despite advances in diagnosis and treatment of CRC, and improved outcomes as a result, there is still a dire need for improvement of patient stratification. It has become clear that patients with *BRAF* mutations have a poor prognosis and are, together with *RAS* mutations, resistant to the effects of anti-EGFR treatment [2, 3]. In addition to the analysis of known (proto) oncogenes such as *RAS/RAF,* patient stratification can also be based on clinical variables, gene expression profiles, or by analysis of the stroma [3–8].

In tumors, stroma is the collective of non-cancer cells and consists of extracellular matrix, endothelial and immune cells, but its main cellular constituents are cancer-associated fibroblasts (CAFs) that can exist in various degrees of activation in response to tumor cell-derived signals. In general, the CAFs are considered to be tumor-promoting but exceptions to this paradigm are now apparent, most notably in pancreatic cancer where both tumor-promoting and tumor-inhibiting signals are produced by the stroma [9].

Matrix metalloproteases (MMPs), a class of matrix-degrading enzymes, are among the key protein families in the stroma associated with tumorigenesis [10]. The protein A Disintegrin And Metalloprotease-12 (ADAM12), is closely related to the MMPs and involved in the remodeling of the extracellular matrix (ECM) and cell signaling through cleavage of ECM and the release of growth factors [11]. ADAM12 is involved in multiple pathological processes and is most extensively known to be upregulated in cancer, where it may be of significant prognostic value [11–17]. Its expression correlates with tumor stage in breast and bladder cancer [18, 19]. A recent study reported on circulating ADAM12 levels in CRC patients but did not investigate its clinical relevance [20]. Importantly, previous work by our group and others has shown that serum levels of ADAM12 can be used as a minimally-invasive readout for the abundance and activation status of CAFs in the stroma of gastrointestinal cancers [21]. In this study we set out to investigate the clinical value of noninvasive (serum) measurements of the stromal compartment through serum ADAM12 in CRC.

## Methods

### Datasets used for expression analysis

For comparisons of tumor versus non-cancer tissue, four gene expression datasets were used: the Illumina beadchip datasets GSE25070 [22] and GSE37182 [23], Agilent array set GSE28000 [24], and RNA-sequencing data from TCGA [25]. Affymetrix array datasets used to delineate the source of ADAM12 expression in tumors include cell-line data (GSE36133 [26], GSE57083 and E-MTAB-783 [27]), sorted cells (GSE39396 [28]), patient tissue (GSE44861 [29] and GS68468 [30]) and microdissected tissue (GSE35602 (Agilent [31]). Correlation of stromal activation markers was performed on the AMC-AJCCII-90 set [5, 32]. PDX data were from E-MTAB-3980 [33]. See also Additional file 1: Table S1.

### Patient samples and study design

Retrospective analysis of the CAIRO2 trial was conducted, this trial has been published previously (patients enrolled between 2003 and 2004, Trial Registration ID: NCT00312000) [34, 35]. In brief, metastatic CRC patients were randomized between treatment with capecitabine, oxaliplatin and bevacizumab (CAPOX-B) with (CBC treatment arm) or without (CB treatment arm) cetuximab. For more details on patients and methods we refer to the original papers [34, 35]. All patients provided a written informed consent for their data to be collected and analyzed for scientific purposes. For 235 (31%) patients of the 755 included in the trial, serum samples and information on *KRAS* and *BRAF* mutation status were available. Serum samples were collected at the start of the study, 39 patients (17%) had received adjuvant chemotherapy prior to blood sampling. Clinical characteristics are shown in Table 1. Updated progression free survival (PFS) and overall survival (OS) data were obtained in June 2020. In the CB group 98 patients (92%) and CBC group 122 patients (95%) had died.

**Table 1:** Baseline characteristics of analyzed cohort dichotomized by ADAM12 levels

Characteristics		ADAM12 low	ADAM12 high	***P-***value
		(***n*** = 74)	(***n*** = 161)	
Mean age (SD)		64.22 (8.14)	62.90 (9.14)	0.288
Gender n (%)	male	44 (59.5)	101 (62.7)	0.738
	female	30 (40.5)	60 (37.3)	
WHO n (%)	0	62 (83.8)	104 (64.6)	0.005
	1	12 (16.2)	56 (34.8)	
	na	0 (0.0)	1 (0.6)	
Primary tumor location n (%)	left	33 (44.6)	65 (40.4)	0.319
	right	16 (21.6)	48 (29.8)	
	rectum	24 (32.4)	41 (25.5)	
	na	1 (1.4)	7 (4.3)	
Metastasis n (%)	synchronous	22 (29.7)	111 (68.9)	< 0.001
	metachronous	52 (70.3)	50 (31.1)	
Prior adjuvant therapy n (%)	no	59 (79.7)	136 (84.5)	0.414
	yes	15 (20.3)	24 (14.9)	
	na	0 (0.0)	1 (0.6)	
Microsatellite status n (%)	MSI	70 (94.6)	149 (92.5)	0.607
	MSS	4 (5.4)	10 (6.2)	
	na	0 (0.0)	2 (1.2)	
*KRAS* n (%)	wild type	40 (54.1)	107 (66.5)	0.093
	mutant	34 (45.9)	54 (33.5)	
*BRAF* n (%)	wild type	68 (91.9)	144 (89.4)	0.726
	mutant	6 (8.1)	17 (10.6)	
Tumor buds n (%)	< 5	21 (28.4)	37 (23.0)	0.312
	5+	40 (54.1)	104 (64.6)	
	na	13 (17.6)	20 (12.4)	

Pearson Chi-squared test used for categorical variables and unpaired t-test used for continuous variables. Unknowns were excluded for testing variables

*n* number of patients, *na* not available, *SD* standard deviation

From the early-stage rectal cancer pilot cohort (*N* = 20, collected at the Amsterdam University Medical Center, location VUmc between 2016 and 2019), plasma samples were collected retrospectively. Inclusion criteria involved early-stage rectal cancer (stage I-III) with or without recurrence and the availability of plasma for analysis. All treatment modalities were included. The samples were collected prior to treatment. Clinicopathological data were obtained from medical records and included age, gender, tumor stage, treatment and recurrence. Collection of material and study design was approved by the Medical Ethical Committee board of the Amsterdam UMC (Number 2017.302/U2020.049). Written informed consent was obtained from all participants of this study. For clinical characteristics see Additional file 1: Table S5.

Reporting is in accordance with the REMARK (REporting recommendations for tumor MARKer prognostic studies) guidelines [36].

### ELISA analysis of serum samples

Serum samples from the CAIRO2 trial and plasma samples from the rectal cancer pilot patient were available for analysis. All samples were stored at − 80 °C until analysis. ADAM12 was measured with the hADAM12 DuoSet ELISA (R&D Systems, Minneapolis, MN). 96-well plates (Nunc MaxiSorp from Greiner, Kremsmünster, Austria) were coated with capture antibody overnight, and blocked with 1% BSA solution the following day. The rectal plasma samples were recalcified by incubation with CaCl_2_ (12 mM) to induce clotting. 50 μl of sample was added for two hours. After mild washing steps, biotinylated detection antibody was added for two hours. This was followed by 20 min incubation with horse-radish peroxidase (HRP)-labeled streptavidin. Tetramethylbenzidine substrate solution (TMB) was added for 20 min, and the reaction stopped using 1 M H_2_SO_4_. Absorbance was measured at 450 nm and 540 nm for serum samples and 450 nm and 570 nm for plasma samples (BioTek Synergy BioTek, Winooski, VT). The 540 nm or 570 nm readings were subtracted from the 450 nm values prior to further analysis.

### CMS classification and tumor budding

We have previously established an immunohistochemical (IHC-)classifier to identify the consensus molecular subtypes (CMSs), and applied this on tissue samples available from the CAIRO2 cohort. CMS labels were retrieved from this publication [37, 38].

Scoring of tumor budding for the CAIRO2 trial was performed previously on pan-cytokeratin-stained tissue microarrays by means of intratumoral budding. Tumor budding status was reported as low or high, using a cut-off of 5 tumor buds, retrieved from the article [39].

### Data analysis and statistics

R was used for expression analysis of ADAM12 in multiple datasets and linear regression analysis of gene expression. The Kaplan-Meier method was used to estimate survival curves and compared by means of the log-rank test for both OS and PFS. We performed uni- and multivariable analysis using a cox proportional hazard regression analysis to investigate the association between survival with ADAM12 groups (univariate), and adjusted for the following variables: gender, age, performance status (WHO), timing of metastasis, having received prior adjuvant therapy, *KRAS* and *BRAF* mutation status and treatment arm (multivariate). Patients with missing data were excluded from the analysis. A *P*-value below 0.05 was considered statistically significant. Statistical tests applied are indicated in figure legends.

## Results

### ADAM12 is upregulated in CRC stroma

**Fig. 1:**
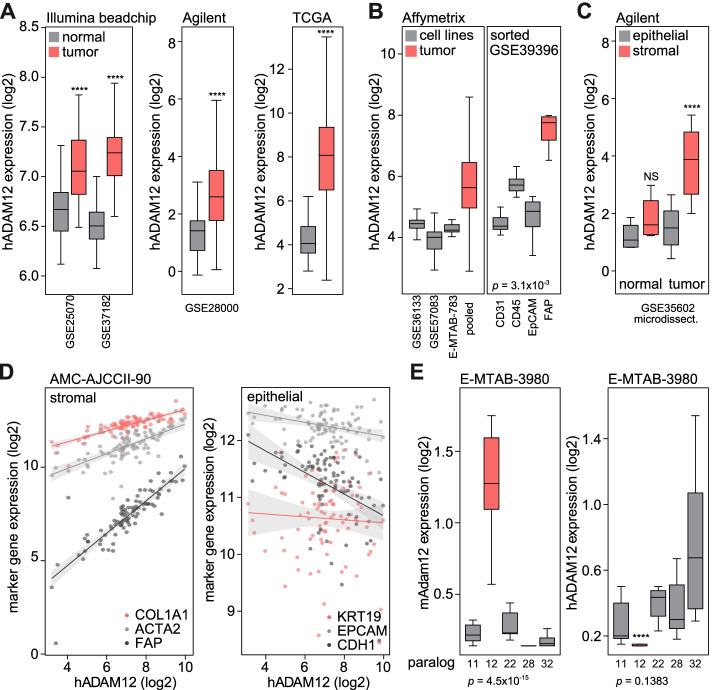
ADAM12 is upregulated in CRC and is expressed in the CRC stroma. Expression levels of ADAM12 are shown in tumor versus normal tissue. All datasets used are indicated in Supplementary Table S1 (**A**). In left panel, expression of ADAM12 in cell lines is compared to pooled Affymetrix data from bulk tumor (GSE44861 and GSE68468). In right panel, showing expression in sorted cell populations from dissociated tumors. CD31, endothelial cells; CD45, immune cells; EpCAM; epithelial (tumor) cells; FAP, (myo) fibroblast marker (**B**). Expression levels of ADAM12 in tumor tissue compared to stromal tissue in microdissected CRC and non-cancerous tissue (**C**). Expression of ADAM12 was correlated to stromal activation markers in the AMC-AJCCII-90 dataset. Collagen type 1 alpha 1 (COL1A1) R2 = 0.6371, *p* < 2.2 × 10–16; α-smooth muscle actin (ACTA2) R2 = 0.4736, *p* = 4.0 × 10–14; Fibroblast Activation Protein (FAP) R2 = 0.7450, p < 2.2 × 10–16. Epithelial markers: cytokeratin 19 (KRT19) R2 = -0.0082, *p* = 0.6014; Epithelial Cell Adhesion Molecule (EPCAM) R2 = 0.1106, *P* = 0.0007; E-cadherin (CDH1) R2 = 0.2123, *p* = 2.9 × 10–6 (**D**). Species specific transcript analysis of ADAM paralogs in patient-derived xenografts was performed. First panel shows mouse reads (Adam), second panel shows human reads (ADAM) (**E**). *P*-value indicated underneath panels is by ANOVA test, comparing conditions within panel. Asterisks indicate significance level tested by T-test between mAdam12 and hADAM12 per dataset

In four publicly available gene expression datasets for CRC and non-cancerous colon tissue, high expression of *ADAM12* was found in tumor tissue (Fig. 1A). Previous work has demonstrated a distinctly stromal expression pattern of *ADAM12* in gastrointestinal tumors [21]. In agreement, we found that *ADAM12* expression was high in CRC bulk tumor tissue but low in pure epithelial cell populations (Fig. 1B). In microdissected cancer tissue, *ADAM12* expression was confined to the tumor stromal fraction (Fig. 1C). Analysis of populations of cells sorted from cancer tissue revealed the Fibroblast Activation Protein (FAP)-positive CAF population to be the predominant source of *ADAM12* expression (Fig. 1B). In our AMC-AJCCII-90 gene expression dataset, *ADAM12* gene expression strongly correlated with markers for (myo) fibroblasts such as *FAP*, Collagen type 1 alpha 1 (*COL1A1*), and α-Smooth Muscle Actin (*ACTA2*) (Fig. 1D). Negative correlations were found with epithelial markers; Cytokeratin 19 (*KRT19*), Epithelial Cell Adhesion Molecule (*EPCAM*), and E-cadherin (*CDH1*). Using the activated and normal stromal gene signatures established in pancreatic cancer by Moffitt et al., *ADAM12* expression was found to associate mostly with activated stroma rather than its abundance per se (Additional file 1: Fig. S1) [40].

Patient-derived xenografts offer the possibility to distinguish host and grafted tumor by species-specific transcript analysis [33, 41]. Expression levels of mouse *Adam* and human *ADAM* paralogs were queried in xenograft-derived datasets (Fig. 1E). In the mouse compartment that represents the stroma, *Adam12* expression was consistently high. Together, these data indicate that ADAM12 is specific to the CAF component of the tumor stroma in CRC.

### High serum ADAM12 levels associate with unfavorable outcome

Human ADAM12 exists as soluble (iso) forms that can be detected in the circulation, and serum levels of this protein can serve as non-invasive proxies for stromal activation in tumor tissue [20, 21, 42]. ADAM12 levels were measured by ELISA in all available serum samples from the CAIRO2 cohort (*n* = 235 (31%)). ADAM12 levels were high with a median of 603 (IQR 1351) pg/mL, compared to previously published healthy controls (median 153 (IQR 169) pg/mL [21]), and higher compared to metastatic esophagogastric adenocarcinoma (median 242.5 pg/mL) [43] and esophageal adenocarcinoma (median 108.7 pg/mL) [44], but lower than metastatic pancreatic ductal adenocarcinoma patients (median 2293 pg/mL) [21].

To investigate a possible connection between stromal activation status and clinical outcome in CRC, patients were stratified into two groups based on serum ADAM12 levels. The optimal cutoff for patient dichotomization was found by assessing the optimal AUC-value of the time-dependent receiver operating characteristic (ROC) curve and maximum Youden index [45, 46]. This was found to be 222 pg/mL at 48 months of follow up.

Baseline characteristics of patients dichotomized by this ADAM12 cutoff show that high levels of ADAM12 were associated with more synchronous metastasis and a lower WHO performance status (Table 1). No differences were observed between the ADAM12 high and ADAM12 low groups with primary tumor location, molecular markers (MSI, *KRAS* and *BRAF* mutational status) or tumor budding.

**Fig. 2:**
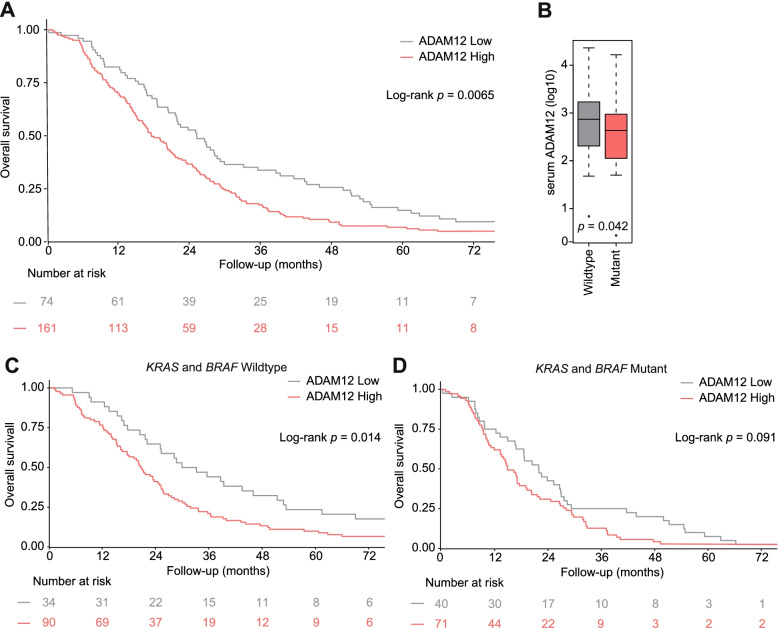
High serum ADAM12 levels associate with unfavorable outcome. **A** Serum levels of ADAM12 were measured by ELISA in 235 patients from the CAIRO2 cohort, and patients in the two trial arms were dichotomized by ADAM12 levels (222 pg/mL). Survival analysis by Kaplan-Meier is shown. **B** Absolute serum levels of ADAM12 were stratified by *KRAS* and *BRAF* mutant or wildtype. Significance is tested by unpaired two tailed Student’s t-test. **C** Kaplan-Meier analysis in the KRAS and BRAF wildtype cohort, dichotomized by serum ADAM12. **D** Kaplan-Meier analysis in the KRAS and BRAF mutant cohort, dichotomized by serum ADAM12

When comparing the two ADAM12 groups, ADAM12 was found to be a prognostic marker for OS (ADAM12 low versus ADAM12 high; median OS (mOS) 25.3 vs. 17.7 months, HR 1.48 [95% CI 1.11–1.96], *P* = 0.007) (Fig. 2A and Additional file 1: Table S2). Even when adjusted for multiple other relevant (prognostic) variables (gender, age, performance status, timing of metastasis, having received prior adjuvant therapy, *RAS*- and *BRAF* mutation status and treatment arm) ADAM12 high was significantly associated with poor outcome (HR 1.42 [95% CI 1.03–1.97], *P* = 0.033) (Additional file 1: Table S2 and Fig. S2). When stratified according to *BRAF* and *KRAS* mutation status, serum ADAM12 levels were higher in wildtype patients (Fig. 2B). Regarding survival, ADAM12 low patients still showed a much better mOS compared to ADAM12 high patients, but was only significant for the *BRAF* and *KRAS* wildtype group (ADAM12 low versus ADAM12 high; mOS 31.6 vs. 20.8 months, HR 1.67 [95% CI 1.10–2.53], *P* = 0.015) (Fig. 2C and D and Additional file 1: Table S2). For progression-free survival ADAM12 was only prognostic in the CBC treatment arm (ADAM12 low versus ADAM12 high; median PFS 11.3 vs. 8.6 months, HR 1.48 [95% CI 1.01–2.19], *P* = 0.047) (Additional file 1: Table S3).

The CAIRO2 trial reported a shorter survival in the experimental arm (CBC) compared to the control (CB) arm, although no selection based on *RAS* mutation status was done as this was not known at the time [34]. When analyzing the association of serum ADAM12 levels with survival between the two trial arms, no difference was observed between the treatment arms in both ADAM12 groups (Additional file 1: Fig. S3 and Table S3). The same was found in both wildtype and mutant population when considering the *BRAF* or *KRAS* mutation status.

### ADAM12 is especially prognostic in rectal tumors

**Fig. 3:**
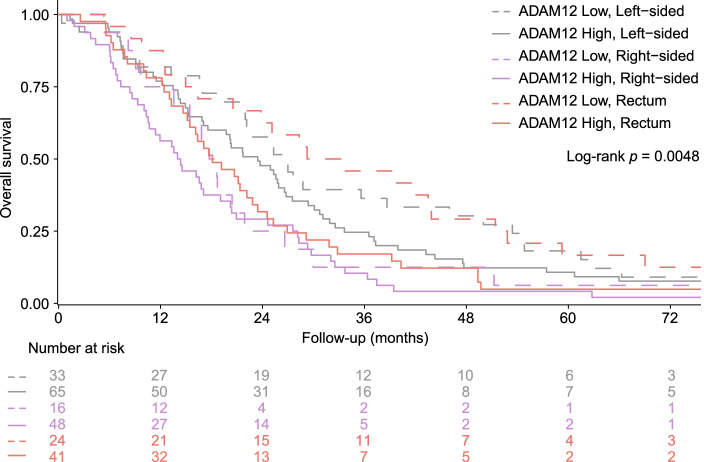
High serum ADAM12 levels associate with unfavorable outcome in tumors located in the rectum. Serum ADAM12 levels were stratified by primary tumor location

It is known that primary tumor location has an impact on prognosis in mCRC [47–51]. We investigated whether there was an association between primary tumor location and serum ADAM12 groups. For 227 (97%) of patients with serum ADAM12 measurements information on primary tumor location was available. Interestingly, the prognostic value of ADAM12 was particularly evident in rectal tumors (ADAM12 low versus ADAM12 high; mOS 31.2 vs. 18.1 months, HR 1.78 [95% CI 1.06–3.00], *P* = 0.030), which remained significant when adjusted for other possible prognostic variables (HR 2.15 [95% CI 1.21–3.82], *P* = 0.009) (Fig. 3 and Additional file 1: Table S4). For left- and right-sided tumors no association with serum ADAM12 and survival was seen.

To further study the prognostic value of serum ADAM12 in rectal tumors specifically, we performed an exploratory analysis in an independent cohort of 20 patients with early-stage and locally advanced rectal tumors (Additional file 1: Table S5). Six patients had detectable ADAM12 levels (median 645 pg/ml), which was significantly associated with a higher stage (stage I/II versus stage III, Mann-Whitney U Test *P* = 0.0287). Four out of eight patients (50%) which developed a recurrence of disease showed high ADAM12 serum levels, compared to two out of ten (20%) without recurrence (recurrence versus no recurrence, Mann-Whitney U Test *P* = 0.168). This shows the potential for serum ADAM12 to serve as a prognostic marker associated with a higher stage and recurrence in local rectal cancer.

### ADAM12 has prognostic potential in mesenchymal tumors

**Fig. 4:**
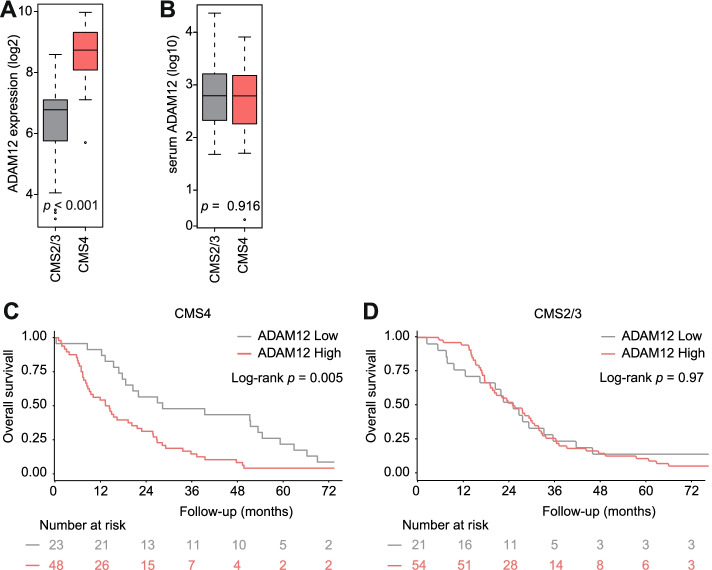
High serum ADAM12 levels associate with unfavorable outcome in mesenchymal tumors. Expression of ADAM12 was stratified by molecular subtypes in the AMC-AJCCII-90 dataset, *N* = 67. Significance is tested by unpaired two tailed Student’s t-test (**A**). Absolute serum levels of ADAM12 in CAIRO2 FFPE samples stratified by molecular subtypes. *N* = 146 (**B**). Serum levels of ADAM12 were dichotomized by ADAM12 levels (222 pg/mL). Survival analysis by Kaplan-Meier is shown for epithelial (CMS2/3) tumors (**C**) and mesenchymal (CMS4) tumors (**D**)

Important phenotypic differences exist between tumors in for instance their growth pattern, therapy resistance, and other features that contribute to poor outcome. This intertumor heterogeneity has been captured and categorized as gene expression based molecular subtypes [52]. In the AMC-AJCCII-90 bulk tumor RNA expression, ADAM12 gene expression was found to be high in CMS4 (Fig. 4A). However, following stratification of the CAIRO2 cohort, using the IHC-classifier for CMS that allows subtype classification of the epithelial (tumor cell) compartment, serum ADAM12 levels were found to be similar between CMS2/3 (*n* = 75) and CMS4 (*n* = 71) tumors (Fig. 4B; CMS1 are MSI tumors and excluded from analysis due to low numbers (*n* = 3)). Moreover, no correlation was found between serum levels of ADAM12 and tumor cellularity (scored by a pathologist) or the keratin positive fraction (epithelium, scored with the image analysis pipeline of the IHC-classifier) (Additional file 1: Fig. S4), suggesting that serum ADAM12 does not associate with stromal abundance per se but specifically informs on its activation status.

Considering the ADAM12 groups within the molecular subtypes, patients with mesenchymal tumors and high ADAM12 have a significant worse PFS and OS as compared to ADAM12 low patients (median PFS 7.15 vs. 12.70 months, HR 1.72 [95% CI 1.027–2.87], *P* = 0.03; mOS 13.8 vs. 28.3 months, HR 2.12 [95% CI 1.25–3.60], *P* = 0.004), OS remained significant when adjusted for the other prognostic variables (HR 2.24 [95% CI 1.07–4.66], *P* = 0.032) (Fig. 4C and Additional file 1: Fig. S5A). For epithelial tumors no correlation between ADAM12 and survival was observed (Fig. 4D and Additional file 1: Fig. S5B). Which might indicate that part of the dismal prognosis of CMS4 tumors can be explained by stromal activation.

## Discussion

There is a paucity of predictive and prognostic stromal markers in CRC, a disease in which the role of the stroma is increasingly evident [28, 53, 54]. In this study, we describe a non-invasive prognostic marker for CAF activity in CRC; ADAM12. Analysis of serum ADAM12 levels in patients enrolled in the CAIRO2 trial showed that highly activated stroma drives unfavorable outcomes, even when corrected for other highly prognostic variables like mutated *BRAF* mutation [55]. The association between high ADAM12 and poor prognosis was remarkably strong in rectal and mesenchymal subtype tumors.

ADAM12 as a prognostic marker has been established in a variety of cancer types, including breast cancer, bladder cancer, lung cancer and ovarian cancer [18, 19, 56–58]. Possible effects of the overexpression of ADAM12 are the upregulation of growth pathways through enhanced expression of a subset of EGFR ligands and increased IGF-1R signaling, which contribute to increased tumor proliferation and metastasis [11, 59]. The poor prognosis in ADAM12-high patients observed in this study may be due to both the high stromal activation and the tumor promoting properties of activation of growth pathways.

To guide optimal treatment strategies, it is important to have information on the prognosis of the patient. For example, triple chemotherapy combined with targeted therapy is an option for fit patients in dire need of aggressive first-line treatment [60]. Serum ADAM12 could be a feasible marker to inform on the aggressiveness of the tumor. The advantage of measuring ADAM12 levels in blood samples rather than tumor biopsies is the non-invasive nature of the measurement. In addition, it circumvents the intratumor heterogeneity that hampers accurate tissue sampling. Although no predictive signal for the addition of cetuximab was found for ADAM12 in the CAIRO2 trial, our recent research has shown that there is possible predictive value for serum ADAM12 in gastrointestinal cancers [21]. Treatment strategies which target the tumor stroma or are dependent on stroma-related properties will most probably be the best candidates for the predictive power of ADAM12. This should be explored in other retrospective cohorts using different treatment strategies.

Tumor-promoting vs -restraining properties have been attributed to the stroma. The intertumor heterogeneity in the stroma has been delineated and roughly two types of CAFs were identified; iCAFS that are characterized by inflammatory programs and cytokine production, and myCAFs that are myofibroblast-like and driven by TGF-b signaling [61]. It is thought that the myCAFs are the tumor-restraining population, and that the iCAF population should be targeted. This is at odds with our finding that ADAM12 (which is produced by CAFs exposed to TGF-b) associates with poor outcome [21]. It is possible that the role of CAFs is disease specific or that myCAFs do in fact harbor tumor-promoting properties. Future analysis of single cell RNA-Seq expression data from the CRC stromal compartment could address this by showing which of the different subsets of CAFs contributes most to the stromal ADAM12, and whether a dichotomization in two classes offers sufficient detail [62].

Another unexpected finding was that ADAM12 serum levels did not associate with mesenchymal subtype (CMS4) tumors in the CAIRO2 cohort using the IHC-classifier, nor with tumor budding [37, 39, 52] Both features are at least in part tumor cell-intrinsic but are also suspected to associate with increased stromal content and activation, respectively. With gene expression data we did see a significant higher expression of *ADAM12* in CMS4 samples in the AMC-AJCCII-90 dataset, but we explain this by the fact that the RNA-based CMS classifier includes the (ADAM12 expressing) stroma, while the IHC-based classification only uses the epithelial compartment of the tumor. CMS4 tumors harbor more stroma, explaining the association in bulk tumor measurements. We take this to imply that ADAM12 is a purely stromal activation marker, as also shown for pancreatic and esophageal cancer [21, 43, 44]. There was however a clear prognostic signal for ADAM12 within the mesenchymal subtype. This might indicate that within these mesenchymal tumors, with abundant stroma, the activation status of the stroma is an important predictor for the aggressiveness of the tumor.

Some limitations of our study should be acknowledged. Serum samples for analysis and *KRAS*/*BRAF* mutation status were only available for 235 (31%) of the 755 patients included in the original CAIRO2 trial. Reassuringly, baseline characteristics of this study population were consistent with the total study population (data not shown). The correlation between serum levels of ADAM12 and tumor tissue gene expression were not assessed, as no gene expression data are available for the CAIRO2 cohort. We currently have no full explanation for the association of ADAM12 high levels with poor prognosis specifically in rectal tumors; this can possibly be explained by a differential stromal recruitment and activation between tumors along the proximal-distal gastrointestinal axis. Furthermore, the reported findings should be validated in another cohort of, preferably untreated, CRC patients. Positive findings from such analyses could be used to design prospective studies that use minimally-invasive assessments of stromal activation to stratify patients.

## Conclusions

In conclusion, in this proof of concept study we have demonstrated that stromal activation in CRC can be monitored in blood samples and that these measurements bear prognostic value. Pending further validation in additional cohorts, blood-borne proxies for stromal activation could function to improve patient’s clinical management.

## Supplementary Information

**Additional file 1: Supplementary Table S1**. Expression dataset used for analysis. **Supplementary Table S2**. Prognostic value of ADAM12. **Supplementary Table S3**. Association of ADAM12 groups with survival, treatment and *KRAS* and *BRAF* mutation status. **Supplementary Table S4**. Prognostic value of ADAM12, stratified by primary tumor location. **Supplementary Table S5**. Characteristics pilot rectal cancer (*n* = 20). **Supplementary Figure S1**. ADAM12 expression correlates with activated stroma signature. **Supplementary Figure S2**. Multivariate analysis of relevant parameters and overall survival. **Supplementary Figure S3**. High serum ADAM12 levels associate with unfavorable outcome independent of treatment status. **Supplementary Figure S4**. Association between ADAM12 serum concentration and percentage tumor epithelium. **Supplementary Figure S5**. High serum ADAM12 levels associate with unfavorable outcome in mesenchymal tumours.

## Data Availability

Data used in this study are available from the corresponding author on reasonable request, only with permission of the authors of the original study.

## Abbreviations

ADAM12: A Disintegrin And Metalloprotease-12
CAFs: cancer-associated fibroblasts
CAPOX-B: Capecitabine, oxaliplatin and bevacizumab
CMS: Consensus molecular subtypes
CRC: Colorectal cancer
ECM: Extracellular matrix
MMPs: Matrix metalloproteases
PFS: Progression free survival
mOS: median overall survival
OS: Overall survival
