# Supplementary material for: Serum-based measurements of stromal activation through ADAM12 associate with poor prognosis in colorectal cancer
Source: BMC Cancer. 2022 Apr 12;22:394. doi: 10.1186/s12885-022-09436-0 (PMC9004139; doi:10.1186/s12885-022-09436-0)
Supplement: Supplementary file 1 — Additional file 1: Supplementary Table S1. Expression dataset used for analysis. Supplementary Table S2. Prognostic value of ADAM12. Supplementary Table S3. Association of ADAM12 groups with survival, treatment and KRAS and BRAF mutation status. Supplementary Table S4. Prognostic value of ADAM12, stratified by primary tumor location. Supplementary Table S5. Characteristics pilot rectal cancer (n = 20). Supplementary Figure S1. ADAM12 expression correlates with activated stroma signature. Supplementary Figure S2. Multivariate analysis of relevant parameters and overall survival. Supplementary Figure S3. High serum ADAM12 levels associate with unfavorable outcome independent of treatment status. Supplementary Figure S4. Association between ADAM12 serum concentration and percentage tumor epithelium. Supplementary Figure S5. High serum ADAM12 levels associate with unfavorable outcome in mesenchymal tumours. [file 12885_2022_9436_MOESM1_ESM.pdf]

Supplementary Table S1. Expression dataset used for analysis

	accession	platform	author	ref	source
Figure 1a	GSE25070	Illumina Beadchip	Hinoue	(22)	tumor and normal
	GSE37182	Illumina Beadchip	Musella	(23)	tumor and normal
	GSE28000	Agilent	Jovov	(24)	tumor and normal
	TCGA-COAD	RNAseq	TCGA	(25)	tumor and normal
Figure 1b	GSE36133	Affymetrix	Barrettina	(26)	cell lines
	GSE57083	Affymetrix	Wappett	-	cell lines
	E-MTAB-783	Affymetrix	Garnett	(27)	cell lines
	GSE44861	Affymetrix	Ryan	(29)	tumor and normal
	GSE68468	Affymetrix	Sheffer	(30)	tumor, normal, and metastatic
	GSE39396	Affymetrix	Calon	(28)	sorted
Figure 1c	GSE35602	Agilent	Nishida	(31)	microdissected
Figure 1d	GSE33114	Affymetrix	De Sousa	(5)	tumor, normal, and cells
Figure 1e	E-MTAB-3980	Illumina RNAseq	Bradford	(33)	patient-derived xenografts

Supplementary Table S2. Prognostic value of ADAM12.

Mutation group	Patients/ Events	Median survival (mo) ADAM12 low	ADAM12 high	Unadjusted HR (95% CI)	*P*-value	Adjusted HR (95% CI)	*P*-value
PFS							
All	235/230	11.7	9.3	1.22 (0.92-1.61)	0.156	1.11 (0.82-1.52)	0.478
KRAS/BRAF Wildtype	124/120	13.1	10.1	1.40 (0.94-2.10)	0.093	1.21 (0.77-1.90)	0.398
KRAS/BRAF Mutant	111/110	9.15	8.3	1.10 (0.75-1.63)	0.627	1.24 (0.79-1.97)	0.351
OS							
All	235/220	25.3	17.7	1.48 (1.11-1.96)	0.007	1.42 (1.03-1.97)	0.033
KRAS/BRAF Wildtype	124/112	31.6	20.8	1.67 (1.10-2.53)	0.015	1.53 (0.94-2.50)	0.090
KRAS/BRAF Mutant	111/108	21.9	14.9	1.41 (0.95-2.10)	0.090	1.56 (0.98-2.47)	0.062

HR Adjusted for sekse, age, WHO status, timing of metastasis, adjuvant chemotherapy, KRAS and BRAF mutation status and treatment arm. HR, hazard ratio; OS, overal survival; PFS, progression free survival

Supplementary Table S3. Association of ADAM12 groups with survival, treatment and *KRAS* and *BRAF* mutation status

					KRAS/BRAF Wildtype				*KRAS/BRAF* Mutant			
Variable	Patients/ events	ADAM12 Low	ADAM12 High	*P*- value	Patients/ events	ADAM12 Low	ADAM12 High	*P*- value	Patients/ events	ADAM12 Low	ADAM12 High	*P*- value
Median PFS (months)												
Total	235/230	11.7	9.3	0.159	124/120	13.1	10.1	0.099	111/110	9.15	8.3	0.617
CB	107/103	12.1	9.6	0.790	57/54	13.1	9.6	0.821	50/49	12.1	9.7	0.428
CBC	128/127	11.3	8.6	**0.046**	67/66	13.1	11.2	**0.020**	61/61	8.3	7.3	0.773
P value		0.370	0.244			0.411	0.276			0.912	0.803	
Median OS (months)												
Total	235/227	25.3	17.7	**0.007**	124/112	31.6	20.8	**0.014**	111/108	21.9	14.9	0.091
CB	107/101	25.3	18.4	0.146	57/50	33.7	20.7	0.151	50/48	16.8	14.9	0.337
CBC	128/126	25.3	17.6	**0.019**	67/62	31.6	20.9	**0.038**	61/60	22.0	15.6	0.170
P value		0.806	0.484			0.712	0.697			0.679	0.618	

CB, capecitabine, oxaliplatin and bevacizumab; CBC, CB with cetuximab; PFS = Progression free survival; OS = Overall survival

Supplementary Table S4. Prognostic value of ADAM12, stratified by primary tumor location

Primary tumor location	Patients/ Events	Median survival (mo) ADAM12 low	ADAM12 high	Unadjusted HR (95% CI)	*P*-value	Adjusted HR (95% CI)	*P*-value
PFS							
Left-sided	98/95	12.2	10.8	1.21 (0.78-1.86)	0.387	1.03 (0.61-1.73)	0.920
Right-sided	64/63	8.35	8.1	0.77 (0.43-1.37)	0.368	0.64 (0.34-1.19)	0.155
Rectum	65/64	13.1	9.3	1.49 (0.89-2.52)	0.126	1.45 (0.84-2.51)	0.178
OS							
Left-sided	98/90	26.9	23.4	1.29 (0.84-2.00)	0.244	1.03 (0.59-1.80)	0.911
Right-sided	64/62	18.2	14.2	1.24 (0.69-2.23)	0.468	1.38 (0.73-2.61)	0.322
Rectum	65/60	31.2	18.1	1.78 (1.06-3.00)	0.030	2.31 (1.28-4.19)	0.006

HR Adjusted for sekse, age, WHO status, timing of metastasis, adjuvant chemotherapy, KRAS and BRAF mutation status and treatment arm. HR, hazard ratio; OS, overal survival; PFS, progression free survival

Supplementary Table S5. Characteristics pilot rectal cancer (n = 20)

Mean age		62.7
Gender	Male	12
	Female	8
Stage	I	11
	IIA	1
	IIIA	2
	IIIB	6
Neoadjuvant	No	12
	Radiotherapy	3
	Radiochemotherapy	5
Treatment	Abdominoperineal resection (APR)	2
	Low anterior resection (LAR)	7
	Transanal Minimally Invasive Surgery (TAMIS)	5
	Transanal total mesorectal excision (TaTME)	6
Recurrence	Yes	8
	No	12

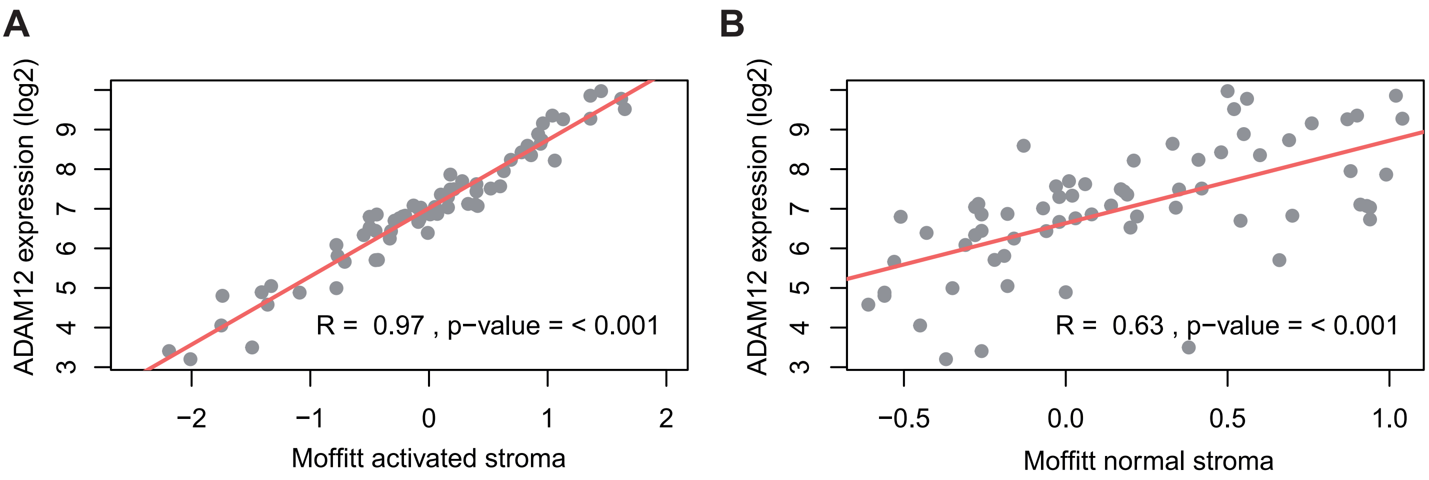

Supplementary Fig. S1 ADAM12 expression correlates with activated stroma signature
Expression of ADAM12 in the AMC-AJCCII-90 dataset was correlated with the Moffitt gene signatures for activated stroma (A) and normal stroma (B). Moffit activated stroma gene signature comprised of the top 24 genes and the Moffitt normal stroma gene signature of the top 23 genes. Correlation was quantified using Pearson's correlation coefficient.

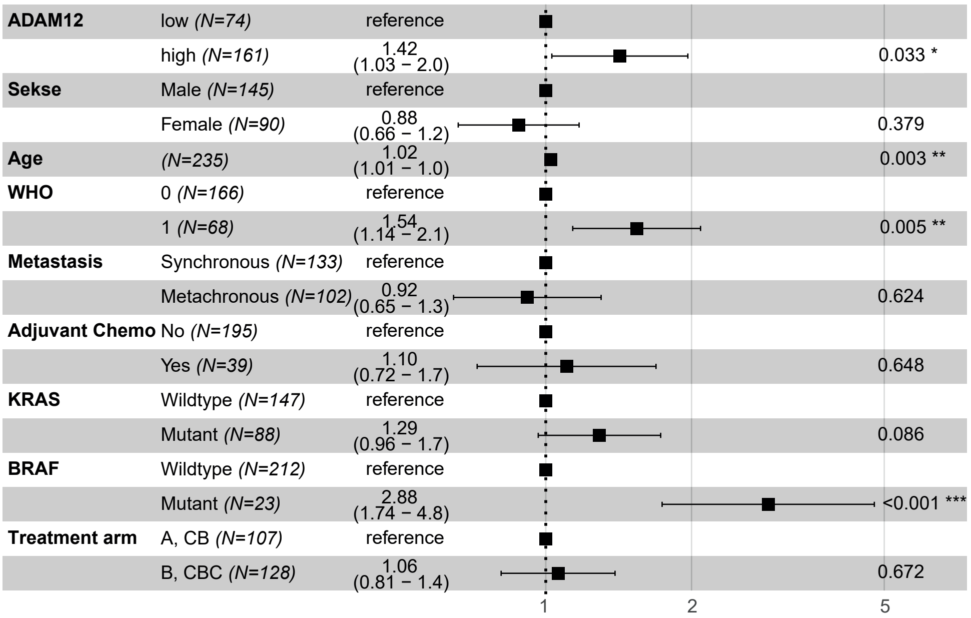

Supplementary Fig. S2 Multivariate analysis of relevant parameters and overall survival.

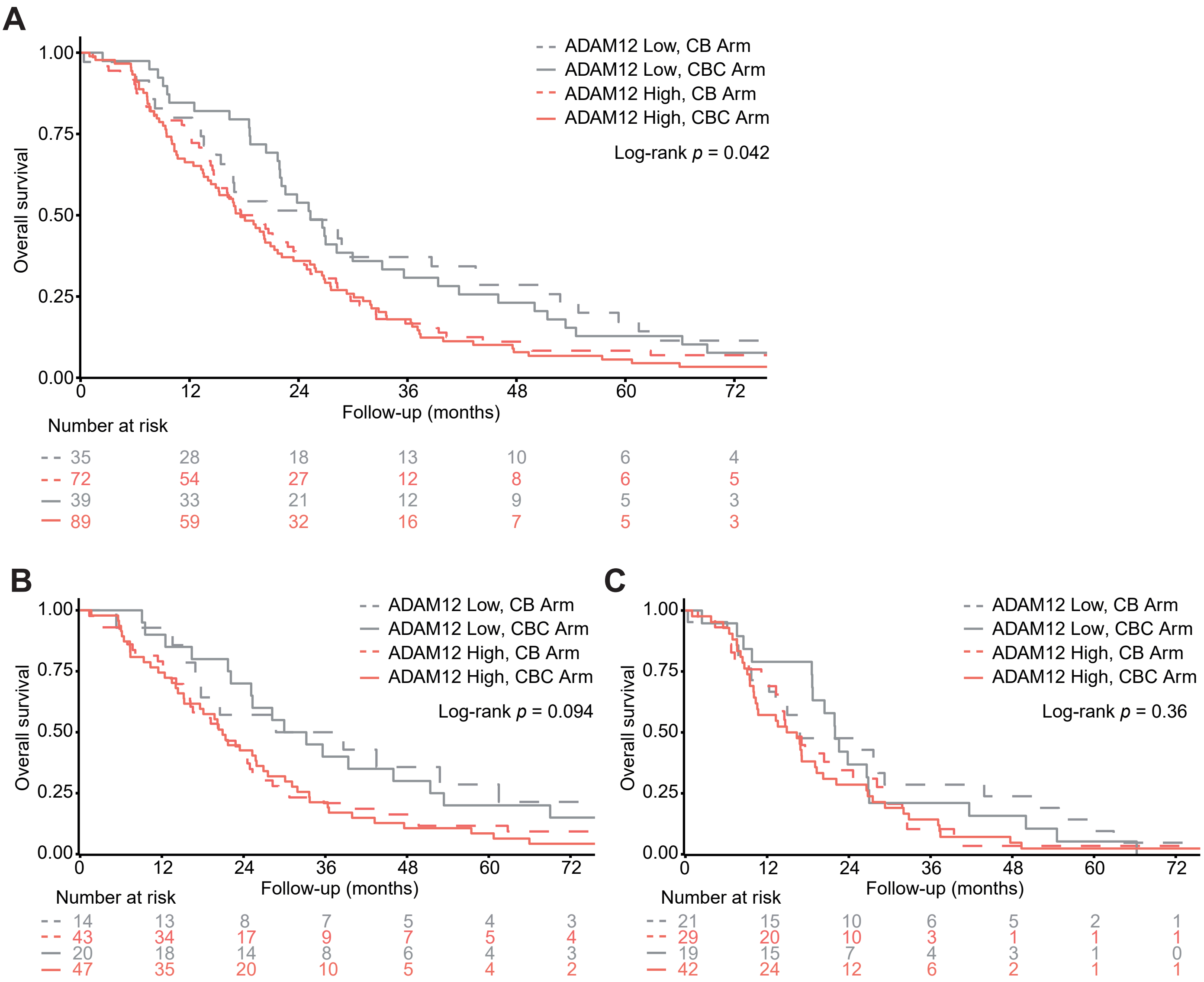

Supplementary Fig. S3 High serum ADAM12 levels associate with unfavorable outcome independent of treatment status.

Serum levels of ADAM12 were measured by ELISA in 235 patients from the CAIRO2 cohort, and patients in the two trial arms were dichotomized by ADAM12 levels (222 pg/mL) and stratified by treatment arm; control arm (CB, capecitabine, oxaliplatin and bevacizumab), experimental arm (CBC, capecitabine, oxaliplatin, bevacizumab and cetuximab). Survival analysis by Kaplan-Meier is shown (A). Kaplan-Meier analysis in the *KRAS* and *BRAF* wildtype cohort (B). Kaplan-Meier analysis in the *KRAS* and *BRAF* mutant cohort (C).

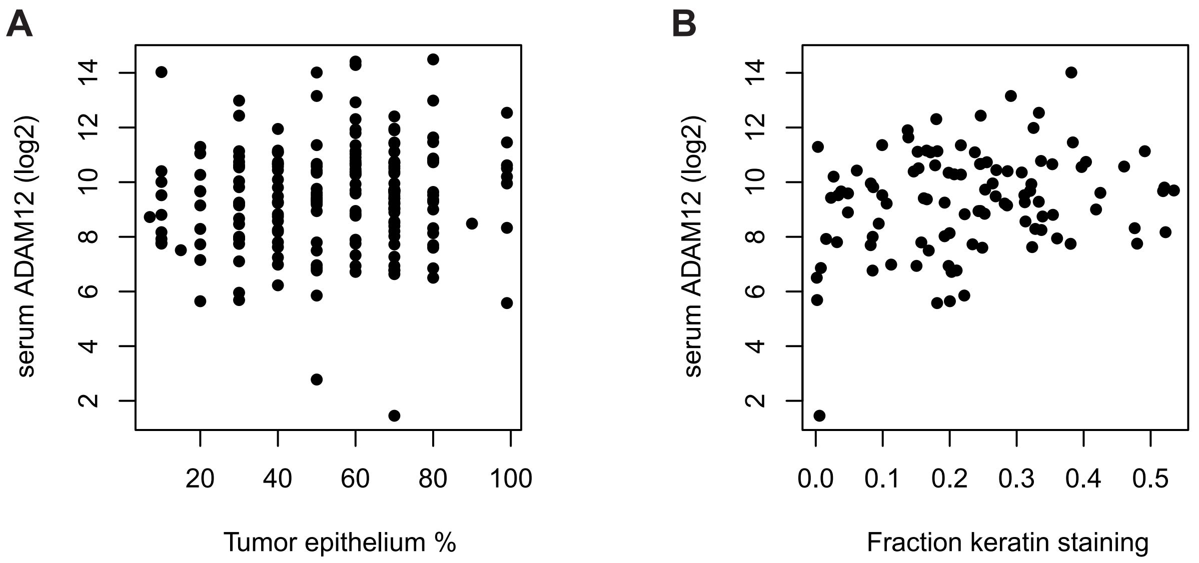

Supplementary Fig. S4 Association between ADAM12 serum concentration and percentage tumor epithelium measured manually (**A**) or with a keratin staining (**B**).

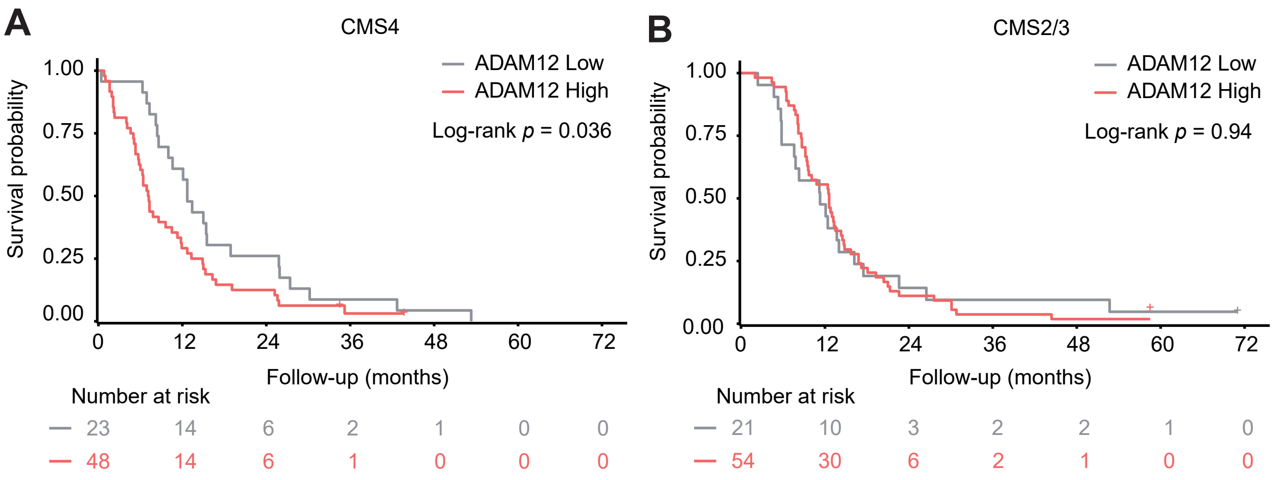

Supplementary Fig. S5 High serum ADAM12 levels associate with unfavorable outcome in mesenchymal tumours.
Serum levels of ADAM12 were dichotomized by ADAM12 levels (222 pg/mL) in CAIRO2 FFPE samples stratified by molecular subtypes. N=146. (**A**) Progression free survival analysis by Kaplan-Meier is shown for epithelial (CMS2/3) tumours and **(B)** mesenchymal (CMS4) tumours.
